# Supplementary material for: Differential effects of HDAC inhibitors in the RhoI255d mouse model for autosomal dominant retinitis pigmentosa
Source: Cell Death Discov. 2025 Dec 14;12:57. doi: 10.1038/s41420-025-02908-9 (PMC12848085; doi:10.1038/s41420-025-02908-9)
Supplement: Supplementary file 1 — supplemental table (pdf) [file 41420_2025_2908_MOESM1_ESM.pdf]

Supplemental Tables

Table S1. Increased HDAC activity in *Rho*$^{I255d/+}$ photoreceptors and application of HDAC inhibitors (TSA and NAM): Quantitative data for graphs presented in Figure 1E.

Table S1 (Figure)	Genotype	*p* - value comparison	Genotype-treatment	Mean ± SD (%)	*p* - value	n
Fig. 1E	WT	$R^{I255d/+}$ - NT	NT	0.44 ± 0.34	$p < 0.0001$	6
			TSA	0.24 ± 0.30	$p < 0.0001$	3
			NAM	0.32 ± 0.35	$p < 0.0001$	3
			TSA+NAM	0.19 ± 0.21	$p < 0.0001$	3
	$R^{I255d/+}$	$R^{I255d/+}$ - NT	NT	3.55 ± 1.39		6
			TSA	1.26 ± 0.30	$p < 0.0001$	6
			NAM	1.97 ± 0.89	$p = 0.0003$	6
			TSA+NAM	0.94 ± 0.52	$p < 0.0001$	6
		$R^{I255d/+}$ - TSA	NAM		$p = 0.0426$	
		$R^{I255d/+}$ - TSA	TSA+NAM		$p = 0.3475$	
		$R^{I255d/+}$ - NAM	TSA+NAM		$p = 0.0049$	

Table S2. Effect of SAHA (S) on cell death (TUNEL assay) in outer nuclear layer (ONL) and inner nuclear layer (INL), and ONL row counts, with concentrations (0.01, 0.1, and 1 µM): Quantitative data for graphs presented in Figures 2B, C, D.

Table S2 (Figure)	Parameter	*p* - value comparison	Genotype-treatment	Mean ± SD (%)	*p* - value	n
Fig. 2B	ONL-TUNEL	WT - NT	WT - NT	2.10 ± 0.79		6
			WT - S 0.01	2.91 ± 0.38	$p = 0.3699$	6
			WT - S 0.1	2.76 ± 0.48	$p = 0.5568$	4
			WT - S 1	9.57 ± 2.54	$p < 0.0001$	3
		$R^{I255d/+}$ - NT	WT - NT		$p < 0.0001$	
			$R^{I255d/+}$ - NT	7.28 ± 1.79		16
			$R^{I255d/+}$ - S 0.01	7.94 ± 0.90	$p = 0.3952$	5
			$R^{I255d/+}$ - S 0.1	3.44 ± 1.50	$p < 0.0001$	10
			$R^{I255d/+}$ - S 1	7.18 ± 1.48	$p = 0.9916$	5
Fig. 2C	INL-TUNEL	WT - NT	WT - NT	0.09 ± 0.09		6
			WT - S 0.01	0.09 ± 0.09	$p = 0.9790$	6
			WT - S 0.1	0.08 ± 0.09	$p = 0.7388$	6
			WT - S 1	0.05 ± 0.09	$p = 0.9816$	3
		$R^{I255d/+}$ - NT	WT - NT		$p = 0.3058$	
			$R^{I255d/+}$ - NT	0.09 ± 0.10		16
			$R^{I255d/+}$ - S 0.01	0.07 ± 0.03	$p = 0.1720$	5
			$R^{I255d/+}$ - S 0.1	0.10 ± 0.09	$p = 0.9523$	10
			$R^{I255d/+}$ - S 1	0.14 ± 0.16	$p = 0.9908$	5
Fig. 2D	ONL rows	WT - NT	WT - NT	7.71 ± 0.60		6
			WT - S 0.01	7.87 ± 0.30	$p = 0.6663$	6
			WT - S 0.1	7.98 ± 0.84	$p = 0.5986$	4
			WT - S 1	5.98 ± 0.74	$p = 0.0003$	3
		$R^{I255d/+}$ - NT	WT - NT		$p < 0.0001$	
			$R^{I255d/+}$ - NT	4.93 ± 0.61		16
			$R^{I255d/+}$ - S 0.01	4.19 ± 0.47	$p = 0.1031$	5
			$R^{I255d/+}$ - S 0.1	5.14 ± 0.74	$p = 0.3583$	6
			$R^{I255d/+}$ - S 1	1.51 ± 0.30	$p < 0.0001$	5

Table S3. Effect of SAHA (S) on caspase-3 (cleaved-caspase-3 staining), calpain-2 (calpain-2 staining) and cone survival (cone arrestin-3 staining) in outer nuclear layer (ONL), with concentrations (0.01, 0.1, and 1 µM): Quantitative data for graphs presented in Figures 3B, D, F.

Table S3 (Figure)	Parameter	*p* - value comparison	Genotype-treatment	Mean ± SD (%)	*p* - value	n
Fig. 3B	Caspase-3	WT - NT	WT - NT	0.22 ± 0.08		7
			WT - S 0.01	0.22 ± 0.07	$p = 0.8660$	6
			WT - S 0.1	0.42 ± 0.24	$p = 0.8916$	3
			WT - S 1	0.38 ± 0.36	$p = 0.9440$	3
		$R^{I255d/+}$ - NT	WT - NT		$p < 0.0001$	
			$R^{I255d/+}$ - NT	2.18 ± 1.35		13
			$R^{I255d/+}$ - S 0.01	2.04 ± 0.49	$p = 0.1635$	5
			$R^{I255d/+}$ - S 0.1	0.47 ± 0.36	$p < 0.0001$	9
			$R^{I255d/+}$ - S 1	0.12 ± 0.09	$p < 0.0001$	4
Fig. 3D	Calpain-2	WT - NT	WT - NT	1.71 ± 0.66		7
			WT - S 0.01	2.12 ± 0.38	$p = 0.3523$	6
			WT - S 0.1	2.24 ± 0.11	$p = 0.2942$	3
			WT - S 1	3.84 ± 0.71	$p = 0.0002$	3
		$R^{I255d/+}$ - NT	WT - NT		$p = 0.0059$	
			$R^{I255d/+}$ - NT	3.18 ± 0.92		15
			$R^{I255d/+}$ - S 0.01	2.94 ± 0.30	$p = 0.7241$	5
			$R^{I255d/+}$ - S 0.1	1.64 ± 0.58	$p = 0.0002$	9
			$R^{I255d/+}$ - S 1	2.10 ± 1.04	$p = 0.1037$	5
Fig. 3F	Cone	WT - NT	WT - NT	16.82 ± 0.82		12
			WT - S 0.01	15.56 ± 1.29	$p = 0.0334$	6
			WT - S 0.1	15.34 ± 0.82	$p = 0.3596$	3
			WT - S 1	11.83 ± 0.30	$p = 0.0001$	3
		$R^{I255d/+}$ - NT	WT - NT		$p < 0.0001$	
			$R^{I255d/+}$ - NT	13.46 ± 2.15		12
			$R^{I255d/+}$ - S 0.01	14.72 ± 0.85	$p = 0.5676$	5
			$R^{I255d/+}$ - S 0.1	12.99 ± 2.61	$p = 0.5129$	7
			$R^{I255d/+}$ - S 1	7.56 ± 0.99	$p < 0.0001$	4

Table S4. Effect of MPT (M) on cell death (TUNEL assay) in outer nuclear layer (ONL) and inner nuclear layer (INL) and ONL row counts, with concentrations (0.1, 1, and 10 µM): Quantitative data for graphs presented in Figures 4B, C, D.

Table S4 (Figure)	Parameter	*p* - value comparison	Genotype-treatment	Mean ± SD (%)	*p* - value	n
Fig. 4B	ONL-TUNEL	$R^{I255d/+}$ - NT	WT - NT	2.93 ± 0.85	$p < 0.0001$	6
			$R^{I255d/+}$ - NT	7.09 ± 1.90		16
			$R^{I255d/+}$ - M 0.1	4.94 ± 1.44	$p = 0.0814$	5
			$R^{I255d/+}$ - M 1	3.60 ± 0.67	$p = 0.0045$	5
			$R^{I255d/+}$ - M 10	9.80 ± 1.69	$p = 0.0065$	4
Fig. 4C	INL-TUNEL	$R^{I255d/+}$ - NT	WT - NT	0.09 ± 0.09	$p = 0.1323$	6
			$R^{I255d/+}$ - NT	0.09 ± 0.10		16
			$R^{I255d/+}$ - M 0.1	0.07 ± 0.08	$p = 0.1151$	5
			$R^{I255d/+}$ - M 1	0.04 ± 0.02	$p = 0.0286$	5
			$R^{I255d/+}$ - M 10	0.14 ± 0.08	$p = 0.9104$	4
Fig. 4D	ONL rows	$R^{I255d/+}$ - NT	WT - NT	7.66 ± 0.51	$p < 0.0001$	6
			$R^{I255d/+}$ - NT	4.91 ± 0.65		16
			$R^{I255d/+}$ - M 0.1	5.49 ± 0.86	$p = 0.0407$	5
			$R^{I255d/+}$ - M 1	5.41 ± 0.62	$p = 0.0652$	5
			$R^{I255d/+}$ - M 10	1.23 ± 0.31	$p < 0.0001$	4

Table S5. Effect of ACY (A) on cell death (TUNEL assay) in outer nuclear layer (ONL) and inner nuclear layer (INL) and ONL row counts, with concentrations (0.01, 0.1, 1, and 10 µM): Quantitative data for graphs presented in Figures 5B, C, D.

Table S5 (Figure)	Parameter	*p* - value comparison	Genotype-treatment	Mean ± SD (%)	*p* - value	n
Fig. 5B	ONL-TUNEL	$R^{I255d/+}$ - NT	WT - NT	2.07 ± 0.78	$p = 0.0439$	6
			$R^{I255d/+}$ - NT	7.17 ± 1.92		16
			$R^{I255d/+}$ - A 0.01	5.63 ± 1.00	$p = 0.6600$	5
			$R^{I255d/+}$ - A 0.1	5.20 ± 0.66	$p = 0.5264$	5
			$R^{I255d/+}$ - A 1	4.45 ± 1.06	$p = 0.3312$	5
			$R^{I255d/+}$ - A 10	25.09 ± 7.91	$p < 0.0001$	5
Fig. 5C	INL-TUNEL	$R^{I255d/+}$ - NT	WT - NT	0.08 ± 0.10	$p = 0.5733$	6
			$R^{I255d/+}$ - NT	0.09 ± 0.10		16
			$R^{I255d/+}$ - A 0.01	0.07 ± 0.05	$p = 0.5474$	5
			$R^{I255d/+}$ - A 0.1	0.01 ± 0.02	$p = 0.3278$	5
			$R^{I255d/+}$ - A 1	0.00 ± 0.00	$p = 0.2949$	5
			$R^{I255d/+}$ - A 10	1.46 ± 0.55	$p < 0.0001$	5
Fig. 5D	ONL rows	$R^{I255d/+}$ - NT	WT - NT	7.73 ± 0.65	$p < 0.0001$	6
			$R^{I255d/+}$ - NT	4.97 ± 0.57		16
			$R^{I255d/+}$ - A 0.01	5.20 ± 0.76	$p = 0.2246$	5
			$R^{I255d/+}$ - A 0.1	5.21 ± 0.25	$p = 0.2121$	5
			$R^{I255d/+}$ - A 1	5.15 ± 0.47	$p = 0.2801$	5
			$R^{I255d/+}$ - A 10	5.84 ± 0.82	$p = 0.0074$	5

Table S6. Effect of NAM (N) on cell death (TUNEL assay) in outer nuclear layer (ONL) and inner nuclear layer (INL) and ONL row counts, with concentrations (20, 200, 1000, and 2000 µM): Quantitative data for graphs presented in Figures 6B, C, D.

Table S6 (Figure)	Parameter	*p* - value comparison	Genotype-treatment	Mean ± SD (%)	*p* - value	n
Fig. 6B	ONL-TUNEL	$R^{I255d/+}$ - NT	WT - NT	2.09 ± 0.78	$p = 0.0002$	6
			$R^{I255d/+}$ - NT	7.27 ± 1.80		15
			$R^{I255d/+}$ - N 20	9.74 ± 1.89	$p = 0.2132$	4
			$R^{I255d/+}$ - N 200	11.97 ± 2.66	$p = 0.0022$	5
			$R^{I255d/+}$ - N 1000	15.79 ± 1.29	$p < 0.0001$	5
			$R^{I255d/+}$ - N 2000	16.37 ± 1.74	$p < 0.0001$	7
Fig. 6C	INL-TUNEL	$R^{I255d/+}$ - NT	WT - NT	0.08 ± 0.10	$p = 0.2043$	6
			$R^{I255d/+}$ - NT	0.09 ± 0.10		15
			$R^{I255d/+}$ - N 20	0.02 ± 0.05	$p = 0.0879$	4
			$R^{I255d/+}$ - N 200	0.06 ± 0.06	$p = 0.1543$	5
			$R^{I255d/+}$ - N 1000	0.12 ± 0.11	$p = 0.7861$	5
			$R^{I255d/+}$ - N 2000	0.01 ± 0.02	$p = 0.0167$	5
Fig. 6D	ONL rows	$R^{I255d/+}$ - NT	WT - NT	7.78 ± 0.74	$p < 0.0001$	6
			$R^{I255d/+}$ - NT	4.96 ± 0.60		15
			$R^{I255d/+}$ - N 20	5.32 ± 0.94	$p = 0.1678$	4
			$R^{I255d/+}$ - N 200	5.39 ± 0.26	$p = 0.1096$	5
			$R^{I255d/+}$ - N 1000	5.41 ± 0.63	$p = 0.0972$	5
			$R^{I255d/+}$ - N 2000	5.37 ± 0.34	$p = 0.1164$	5

Table S7. Effect of different HDAC inhibitors on HDAC activity (*in situ* HDAC activity assay) in outer nuclear layer (ONL). Treatments with SAHA (S) at 0.01 and 0.1 µM, MPT (M) at 0.1 and 1 µM, ACY (A) at 0.01, 0.1 and 1 µM, and NAM (N) at 20, 200, 1000, and 2000 µM: Quantitative data for graphs presented in Figures S1B, D, F, H.

Table S7 (Figure)	Treatment	*p* - value comparison	Genotype-treatment	Mean ± SD (%)	*p* - value	n
Fig. S1B	SAHA	$R^{I255d/+}$ - NT	WT - NT	0.51 ± 0.13	*p* = 0.0003	5
			$R^{I255d/+}$ - NT	2.15 ± 0.83		6
			$R^{I255d/+}$ - S 0.01	1.13 ± 0.32	*p* = 0.0024	5
			$R^{I255d/+}$ - S 0.1	0.71 ± 0.23	*p* = 0.0005	6
Fig. S1D	MPT	$R^{I255d/+}$ - NT	WT - NT	0.37 ± 0.12	*p* < 0.0001	4
			$R^{I255d/+}$ - NT	2.34 ± 0.70		6
			$R^{I255d/+}$ - M 0.1	1.61 ± 0.27	*p* = 0.0237	5
			$R^{I255d/+}$ - M 1	1.72 ± 0.40	*p* = 0.0483	5
Fig. S1F	ACY	$R^{I255d/+}$ - NT	WT - NT	0.47 ± 0.13	*p* < 0.0001	4
			$R^{I255d/+}$ - NT	2.39 ± 0.71		6
			$R^{I255d/+}$ - A 0.01	1.48 ± 0.24	*p* = 0.0046	5
			$R^{I255d/+}$ - A 0.1	1.03 ± 0.26	*p* = 0.0001	5
			$R^{I255d/+}$ - A 1	0.79 ± 0.19	*p* < 0.0001	5
Fig. S1H	NAM	$R^{I255d/+}$ - NT	WT - NT	0.48 ± 0.14	*p* = 0.0396	4
			$R^{I255d/+}$ - NT	2.19 ± 0.78		6
			$R^{I255d/+}$ - N 20	1.39 ± 0.40	*p* = 0.3438	4
			$R^{I255d/+}$ - N 200	3.29 ± 0.75	*p* = 0.1347	5
			$R^{I255d/+}$ - N 1000	4.04 ± 0.84	*p* = 0.0125	5
			$R^{I255d/+}$ - N 2000	4.36 ± 3.09	*p* = 0.1168	6

Table S8. Effect of MPT (M) on caspase-3 (cleaved-caspase-3 staining), calpain-2 (calpain-2 staining) and cone survival (cone arrestin-3 staining) in outer nuclear layer (ONL), with concentrations (0.1, 1, and 10 μM): Quantitative data for graphs presented in Figures S2B, D, F.

Table S8 (Figure)	Parameter	*p* - value comparison	Genotype-treatment	Mean ± SD (%)	*p* - value	n
Fig. S2B	Caspase-3	$R^{I255d/+}$ - NT	WT - NT	0.20 ± 0.10	*p* = 0.0004	6
			$R^{I255d/+}$ - NT	2.09 ± 1.46		12
			$R^{I255d/+}$ - M 0.1	2.31 ± 1.02	*p* = 0.2800	5
			$R^{I255d/+}$ - M 1	1.75 ± 0.79	*p* = 0.0661	5
			$R^{I255d/+}$ - M 10	3.74 ± 0.59	*p* = 0.3958	4
Fig. S2D	Calpain-2	$R^{I255d/+}$ - NT	WT - NT	1.53 ± 0.71	*p* = 0.0144	7
			$R^{I255d/+}$ - NT	2.88 ± 0.85		15
			$R^{I255d/+}$ - M 0.1	2.75 ± 0.56	*p* = 0.6988	5
			$R^{I255d/+}$ - M 1	2.45 ± 0.62	*p* = 0.3375	5
			$R^{I255d/+}$ - M 10	5.08 ± 1.63	*p* = 0.0013	4
Fig. S2F	Cone	$R^{I255d/+}$ - NT	WT - NT	16.79 ± 0.81	*p* = 0.0069	11
			$R^{I255d/+}$ - NT	13.05 ± 1.99		13
			$R^{I255d/+}$ - M 0.1	14.83 ± 0.31	*p* = 0.0095	5
			$R^{I255d/+}$ - M 1	16.09 ± 1.14	*p* = 0.0007	5
			$R^{I255d/+}$ - M 10	8.79 ± 1.33	*p* = 0.0035	4

Table S9. Effect of ACY (A) on caspase-3 (cleaved-caspase-3 staining), calpain-2 (calpain-2 staining) and cone survival (cone arrestin-3 staining) in outer nuclear layer (ONL), with concentrations (0.01, 0.1, 1, and 10 μM): Quantitative data for graphs presented in Figures S3B, D, F.

Table S9 (Figure)	Parameter	*p* - value comparison	Genotype-treatment	Mean ± SD (%)	*p* - value	n
Fig. S3B	Caspase-3	$R^{I255d/+}$ - NT	WT - NT	0.20 ± 0.08	p = 0.0026	7
			$R^{I255d/+}$ - NT	2.05 ± 1.39		13
			$R^{I255d/+}$ - A 0.01	0.54 ± 0.16	p = 0.0063	5
			$R^{I255d/+}$ - A 0.1	0.34 ± 0.12	p = 0.0034	5
			$R^{I255d/+}$ - A 1	1.40 ± 0.57	p = 0.0752	5
			$R^{I255d/+}$ - A 10	7.81 ± 2.12	$p < 0.0001$	11
Fig. S3D	Calpain-2	$R^{I255d/+}$ - NT	WT - NT	1.72 ± 0.70	p = 0.0076	6
			$R^{I255d/+}$ - NT	2.90 ± 0.89		14
			$R^{I255d/+}$ - A 0.01	2.74 ± 0.39	p = 0.7271	5
			$R^{I255d/+}$ - A 0.1	2.61 ± 0.67	p = 0.5080	5
			$R^{I255d/+}$ - A 1	2.83 ± 0.76	p = 0.9282	5
			$R^{I255d/+}$ - A 10	0.61 ± 0.44	$p < 0.0001$	5
Fig. S3F	Cone	$R^{I255d/+}$ - NT	WT - NT	16.78 ± 0.81	$p < 0.0001$	11
			$R^{I255d/+}$ - NT	13.44 ± 2.04		13
			$R^{I255d/+}$ - A 0.01	14.51 ± 1.12	p = 0.0508	5
			$R^{I255d/+}$ - A 0.1	16.22 ± 1.71	p = 0.0022	5
			$R^{I255d/+}$ - A 1	15.48 ± 2.14	p = 0.0047	5
			$R^{I255d/+}$ - A 10	13.34 ± 1.67	p = 0.4482	5

Table S10. Effect of NAM (N) on caspase-3 (cleaved-caspase-3 staining), calpain-2 (calpain-2 staining) and cone survival (cone arrestin-3 staining) in outer nuclear layer (ONL), with concentrations (20, 200, 1000, and 2000 µM): Quantitative data for graphs presented in Figures S4B, D, F.

Table S10 (Figure)	Parameter	*p* - value comparison	Genotype-treatment	Mean ± SD (%)	*p* - value	n
Fig. S4B	Caspase-3	$R^{I255d/+}$ - NT	WT - NT	0.24 ± 0.07	$p = 0.0177$	6
			$R^{I255d/+}$ - NT	2.10 ± 1.44		12
			$R^{I255d/+}$ - N 20	2.41 ± 0.84	$p = 0.8119$	4
			$R^{I255d/+}$ - N 200	0.70 ± 0.34	$p = 0.1639$	5
			$R^{I255d/+}$ - N 1000	1.52 ± 0.70	$p = 0.4887$	5
			$R^{I255d/+}$ - N 2000	6.83 ± 3.02	$p = 0.0015$	5
Fig. S4D	Calpain-2	$R^{I255d/+}$ - NT	WT - NT	1.57 ± 0.78	$p = 0.0020$	6
			$R^{I255d/+}$ - NT	3.05 ± 0.83		14
			$R^{I255d/+}$ - N 20	3.13 ± 0.93	$p = 0.8399$	4
			$R^{I255d/+}$ - N 200	2.01 ± 0.28	$p = 0.0796$	5
			$R^{I255d/+}$ - N 1000	2.79 ± 0.38	$p = 0.4842$	5
			$R^{I255d/+}$ - N 2000	3.31 ± 1.00	$p = 0.1981$	6
Fig. S4F	Cone	$R^{I255d/+}$ - NT	WT - NT	16.81 ± 0.81	$p = 0.0206$	11
			$R^{I255d/+}$ - NT	13.21 ± 2.02		12
			$R^{I255d/+}$ - N 20	15.25 ± 0.76	$p = 0.4258$	4
			$R^{I255d/+}$ - N 200	14.26 ± 0.95	$p = 0.6920$	5
			$R^{I255d/+}$ - N 1000	13.60 ± 1.67	$p = 0.2507$	5
			$R^{I255d/+}$ - N 2000	14.91 ± 0.64	$p = 0.7254$	6

Table S11. Assessment of photoreceptor proliferation using bromodeoxyuridine (BrdU) treatment from post-natal day (P) 16-18, combined with HDAC inhibitors treatments from P14-P20, including SAHA (S) at 0.1 µM, MPT (M) at 1 µM, ACY (A) at 10 µM, and NAM (N) at 2000 µM: Quantitative data for graphs presented in Figure S5B.

Table S11 (Figure)	Parameter	*p* - value comparison	Genotype-treatment	Mean ± SD (%)	*p* - value	n
Fig. S5B	BrdU	$R^{I255d/+}$ - NT	WT - NT	0.00 ± 0.00	$p = 0.7721$	6
			$R^{I255d/+}$ - NT	0.00 ± 0.00		5
			$R^{I255d/+}$ - S 0.1	0.12 ± 0.02	$p = 0.9631$	4
			$R^{I255d/+}$ - M 1	0.02 ± 0.05	$p = 0.8423$	4
			$R^{I255d/+}$ - A 10	1.22 ± 1.23	$p = 0.0012$	6
			$R^{I255d/+}$ - N 2000	0.00 ± 0.00	$p = 0.7926$	4
